# Copper acetate-facilitated transfer-free growth of high-quality graphene for hydrovoltaic generators

**DOI:** 10.1093/nsr/nwab169

**Published:** 2021-09-08

**Authors:** Jingyuan Shan, Sunmiao Fang, Wendong Wang, Wen Zhao, Rui Zhang, Bingzhi Liu, Li Lin, Bei Jiang, Haina Ci, Ruojuan Liu, Wen Wang, Xiaoqin Yang, Wenyue Guo, Mark H Rümmeli, Wanlin Guo, Jingyu Sun, Zhongfan Liu

**Affiliations:** Center for Nanochemistry (CNC), Beijing Science and Engineering Center for Nanocarbons, Beijing National Laboratory for Molecular Sciences, College of Chemistry and Molecular Engineering, Peking University, Beijing 100871, China; Academy for Advanced Interdisciplinary Studies, Peking University, Beijing 100871, China; Key Laboratory for Intelligent Nano Materials and Devices of the Ministry of Education, State Key Laboratory of Mechanics and Control of Mechanical Structures, Institute of Nanoscience, Nanjing University of Aeronautics and Astronautics, Nanjing 210016, China; Department of Physics and Astronomy, University of Manchester, Manchester M13 9PL, UK; School of Materials Science and Engineering, China University of Petroleum (East China), Qingdao 266580, China; Department of Physics and Astronomy, University of Manchester, Manchester M13 9PL, UK; College of Energy, Soochow Institute for Energy and Materials Innovations (SIEMIS), Jiangsu Provincial Key Laboratory for Advanced Carbon Materials and Wearable Energy Technologies, Soochow University, Suzhou 215006, China; Beijing Graphene Institute (BGI), Beijing 100095, China; Department of Physics and Astronomy, University of Manchester, Manchester M13 9PL, UK; Center for Nanochemistry (CNC), Beijing Science and Engineering Center for Nanocarbons, Beijing National Laboratory for Molecular Sciences, College of Chemistry and Molecular Engineering, Peking University, Beijing 100871, China; College of Energy, Soochow Institute for Energy and Materials Innovations (SIEMIS), Jiangsu Provincial Key Laboratory for Advanced Carbon Materials and Wearable Energy Technologies, Soochow University, Suzhou 215006, China; Beijing Graphene Institute (BGI), Beijing 100095, China; Center for Nanochemistry (CNC), Beijing Science and Engineering Center for Nanocarbons, Beijing National Laboratory for Molecular Sciences, College of Chemistry and Molecular Engineering, Peking University, Beijing 100871, China; Beijing Graphene Institute (BGI), Beijing 100095, China; College of Energy, Soochow Institute for Energy and Materials Innovations (SIEMIS), Jiangsu Provincial Key Laboratory for Advanced Carbon Materials and Wearable Energy Technologies, Soochow University, Suzhou 215006, China; School of Materials Science and Engineering, China University of Petroleum (East China), Qingdao 266580, China; College of Energy, Soochow Institute for Energy and Materials Innovations (SIEMIS), Jiangsu Provincial Key Laboratory for Advanced Carbon Materials and Wearable Energy Technologies, Soochow University, Suzhou 215006, China; Beijing Graphene Institute (BGI), Beijing 100095, China; Key Laboratory for Intelligent Nano Materials and Devices of the Ministry of Education, State Key Laboratory of Mechanics and Control of Mechanical Structures, Institute of Nanoscience, Nanjing University of Aeronautics and Astronautics, Nanjing 210016, China; College of Energy, Soochow Institute for Energy and Materials Innovations (SIEMIS), Jiangsu Provincial Key Laboratory for Advanced Carbon Materials and Wearable Energy Technologies, Soochow University, Suzhou 215006, China; Beijing Graphene Institute (BGI), Beijing 100095, China; Center for Nanochemistry (CNC), Beijing Science and Engineering Center for Nanocarbons, Beijing National Laboratory for Molecular Sciences, College of Chemistry and Molecular Engineering, Peking University, Beijing 100871, China; Beijing Graphene Institute (BGI), Beijing 100095, China

**Keywords:** chemical vapor deposition, graphene, copper acetate, hydrovoltaic electricity generator, transfer-free growth

## Abstract

Direct synthesis of high-quality graphene on dielectric substrates without a transfer process is of vital importance for a variety of applications. Current strategies for boosting high-quality graphene growth, such as remote metal catalyzation, are limited by poor performance with respect to the release of metal catalysts and hence suffer from a problem with metal residues. Herein, we report an effective approach that utilizes a metal-containing species, copper acetate, to continuously supply copper clusters in a gaseous form to aid transfer-free growth of graphene over a wafer scale. The thus-derived graphene films were found to show reduced multilayer density and improved electrical performance and exhibited a carrier mobility of 8500 cm^2^ V^−1^ s^−1^. Furthermore, droplet-based hydrovoltaic electricity generator devices based on directly grown graphene were found to exhibit robust voltage output and long cyclic stability, in stark contrast to their counterparts based on transferred graphene, demonstrating the potential for emerging energy harvesting applications. The work presented here offers a promising solution to organize the metal catalytic booster toward transfer-free synthesis of high-quality graphene and enable smart energy generation.

## Teaser

An effective approach by utilizing metal-containing species, copper acetate, to catalyze the transfer-free graphene growth over dielectric substrates is put forward. Droplet-based hydrovoltaic electricity generator devices based on thus-grown graphene manifest robust voltage output and long cyclic stability.

## INTRODUCTION

Chemical vapor deposition (CVD) has been recognized as one of the most promising methodologies for the controllable preparation of high-quality graphene. It predominantly uses metals such as Cu as growth supports [1–4]. The transfer process of graphene from metals to insulators is an inevitable and cumbersome step to realize a variety of practical applications and often leads to film damage, corrugation and polymeric contamination, which are ultimately unfavorable for mass production [5]. In view of this, the direct synthesis of graphene on dielectric substrates is of paramount importance toward practical aims, bypassing the transfer procedure. However, directly grown graphene films tend to suffer from low crystal quality, abundant structural defects and concurrent formation of multilayers, resulting in poor electrical/optical properties, which impede high-end applications [6–8].

To address these issues, strategies that use metals for the transfer-free synthesis of graphene have attracted particular interest. Among various strategies aimed toward this purpose, the use of sacrificial metal layers (e.g. Cu, Ni and Ge) has garnered considerable attention [9–11]. However, challenges such as metal removal by evaporation or chemical etching are highly likely to cause metal contamination; as a consequence, the thus-grown graphene loses its fine uniformity. Further attempts such as the introduction of floating metal vapor (e.g. Cu and Ga) derived from metal foil placed upstream or upon the target substrate have been reported [12–14]. However, it is difficult to tailor the sublimation amount of metal vapor, further resulting in metal residues. Recently, an effective maneuver in this direction lies in the utilization of metal-containing species that decompose into metal clusters to aid carbon source cracking at the forefront of graphene growth [15,16]. The concentration of the metal clusters can be well-controlled. Nevertheless, direct graphene formation over insulators based on this approach remains largely unexplored.

Taking the above considerations into account, we herein report the Cu(OAc)_2_-facilitated growth of transfer-free graphene on wafer-scale insulators. Cu(OAc)_2_ was subjected to volatilization using an independent heating system to enable the delivery of Cu clusters. The derived Cu clusters enable effective decomposition of the CH_4_ precursor by reducing its activation energy, as exemplified by density functional theory (DFT) calculations. Thus-grown graphene was found to be of high quality, multilayer-deficient and free of Cu residues. In addition, the yielded transfer-free films were found to exhibit favorable electrical performance, harvesting a carrier mobility of 8500 cm^2^ V^–1^ s^–1^ at room temperature; this value is comparable to that of graphene grown on Cu [16]. As a proof-of-concept, we demonstrated the fabrication of a hydrovoltaic electricity generator device using our transfer-free graphene. The as-constructed generator showed superior voltage output and cyclic stability compared to its transfer-involving graphene counterparts, holding promise for practical applications.

## RESULTS AND DISCUSSION

**Figure 1.**
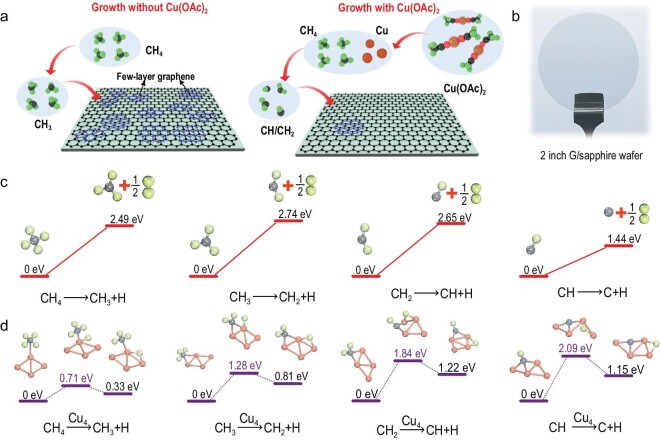
Graphene growth without/with the presence of Cu(OAc)_2_. (a) Schematic illustration of the graphene growth process without/with the presence of Cu(OAc)_2_. (b) Photograph of a 2-inch graphene/sapphire wafer. (c and d) DFT calculation of energy barriers of CH_4_ dehydrogenation in the gas phase (c) without and (d) with Cu clusters.

The growth processes of graphene films without/with the presence of Cu(OAc)_2_ are schematically illustrated in Fig. 1a. In a typical direct CVD system, the carbon source (CH_4_ herein) decomposes into active carbon fragments at elevated growth temperatures (∼1080°C). These active carbon fragments are subsequently adsorbed onto the substrate surface and initiate the formation of graphene domains [17]. The decomposition is normally insufficient because of the catalytic inertness of insulating substrates, resulting in poor crystallinity and massive multilayer formation [18]. By contrast, a floating Cu catalyst can be generated by introducing Cu(OAc)_2_ using an independent heating system. The temperature of the heater (150°C herein) must be maintained below the pyrolysis point of Cu(OAc)_2_ to ensure the *in situ* generation of Cu clusters in the growth zone (Figs S1–S3, Supporting Information). The thus-derived Cu clusters enable effective CH_4_ decomposition by reducing the activation energy, and further induce the facile linkage of *sp*^2^ carbon networks and suppress the formation of amorphous carbon [19]. Notably, graphene grown in the presence of Cu(OAc)_2_ showed a higher percentage of single-layer coverage (black) and a lower percentage of few/multilayer regions (dark blue), whereas graphene without Cu(OAc)_2_ possessed ample multilayer flakes originating from amorphous carbon impurities [20]. This was experimentally verified by scanning electron microscopy (SEM) observations of the typical growth results (Fig. S4). Further, the visible-light transmittance of the as-grown graphene with Cu(OAc)_2_ was found to reach ∼97% (at 550 nm), with dual-side polished quartz or sapphire as the growth substrate. Figure 1b shows a photograph of a 2-inch graphene/sapphire wafer grown via the Cu(OAc)_2_-facilitated route, which showed high transparency and uniformity. Such graphene wafers could be synthesized in a batch-to-batch manner, allowing the production of 10 pieces of 2-inch product per day.

To gain fundamental insight into Cu cluster assistance, decomposition of methane in the absence/presence of Cu(OAc)_2_ was analyzed using DFT calculations [21–25]. Typically, complete decomposition of CH_4_ involves four steps. In the absence of Cu(OAc)_2_, the activation energy for each step reaction was calculated to be 2.49 eV for step I, 2.74 eV for step II, 2.65 eV for step III and 1.44 eV for step IV (Fig. 1c). In contrast, aided by the Cu clusters (here Cu_4_ clusters were selected for modeling), the threshold barrier for CH_4_ decomposition into CH_3_ was calculated to be 0.71 eV, which is markedly lower than that without Cu(OAc)_2_. Evidently, CH_4_ decomposes more completely with the assistance of Cu clusters provided by Cu(OAc)_2_; this is ultimately reflected in the subsequent steps II, III and IV (Fig. 1d). Therefore, an efficient Cu catalyst from Cu(OAc)_2_ could promote decomposition of carbon species in the gas phase to generate highly dehydrogenated CH_x_ active species. Such enhanced decomposition of the gaseous carbon species enables a decrease in the formation probability of *sp*^3^ hybridized carbon, thereby suppressing the few-layer and multilayer regions.

**Figure 2.**
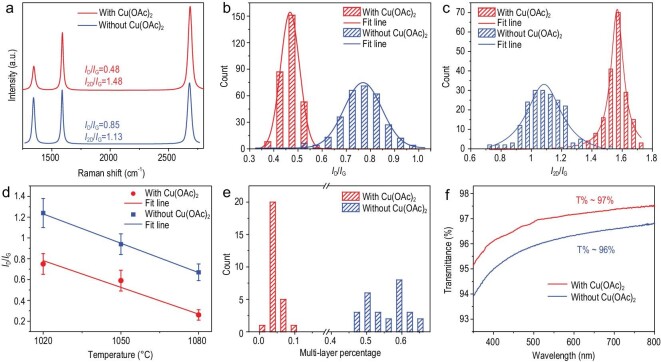
Characterization of transfer-free graphene grown without/with the presence of Cu(OAc)_2_. (a–c) Representative Raman spectra and statistics of the *I*_D_/*I*_G_ and *I*_2D_/*I*_G_ of graphene grown with/without Cu(OAc)_2_. (d) *I*_D_/*I*_G_ variation against the growth temperature of graphene with/without Cu(OAc)_2_. (e) Multilayer percentage of graphene grown with and without Cu(OAc)_2_. (f) UV-vis transmittance spectra of graphene films with/without Cu(OAc)_2_.

The obtained transfer-free graphene films were subjected to various characterization studies to verify the advancement of Cu(OAc)_2_-facilitated growth. Figure 2a shows the representative Raman spectra of the as-grown graphene transferred onto a SiO_2_/Si support (Fig. S5); typical graphene signals including the D peak (∼1350 cm^−1^), G peak (∼1580 cm^−1^) and 2D peak (2700 cm^−1^) can be clearly observed. The intensity ratio of the D and G peaks (*I*_D_/*I*_G_) reflects the defect density of graphene, while *I*_2D_/*I*_G_ reflects the film thickness [26–29]. As such, the *I*_D_/*I*_G_ of graphene prepared by the Cu(OAc)_2_-facilitated process is 0.41, which is markedly lower than that (0.85) of graphene prepared without Cu(OAc)_2_, indicating a reduction in defect density and improvement in film quality and purity. Meanwhile, *I*_2D_/*I*_G_ varies from 1.13 to 1.45 in the presence of Cu(OAc)_2_, suggesting the decreased layer thickness of transfer-free graphene. Raman mapping was further performed to evaluate the uniformity of the graphene film (Figs S6 and S7). As plotted in Fig. 2b and c, the histograms acquired over a survey area of 30 × 30 μm^2^ reflect a significant reduction in *I*_D_/*I*_G_ and a conspicuous increase in *I*_2D_/*I*_G_ with the Cu(OAc)_2_ facilitated route. Samples prior to the transfer process showed similar results (Fig. S8). In addition, this method presents its universality on insulator substrates (Fig. S9). Notably, similar trends can be observed by synthesizing transfer-free graphene at different growth temperatures, ranging from 1020°C through 1050°C to 1080°C (Fig. 2d). The reduced average layer thickness of graphene was derived from the suppressed multilayer graphene regions. A systematic statistical analysis of the percentage of multilayer graphene regions in the entire SEM imaging region without/with the presence of Cu(OAc)_2_ was conducted. As presented in Fig. 2e, the histograms acquired from the statistical results show a significant reduction in the multilayer percentage with the Cu(OAc)_2_ facilitated route. As a result of the reduced layer thickness of transfer-free graphene, the visible-light transmittance (at 550 nm) of the grown graphene (deducting the substrate background) with Cu(OAc)_2_ was improved from 96% to 97% (Fig. 2f). This further suggests that the involvement of a Cu clustering catalyst via Cu(OAc)_2_ is beneficial for mitigating the formation of multilayer domains. These data corroborate the marked improvement in the quality of graphene.

**Figure 3.**
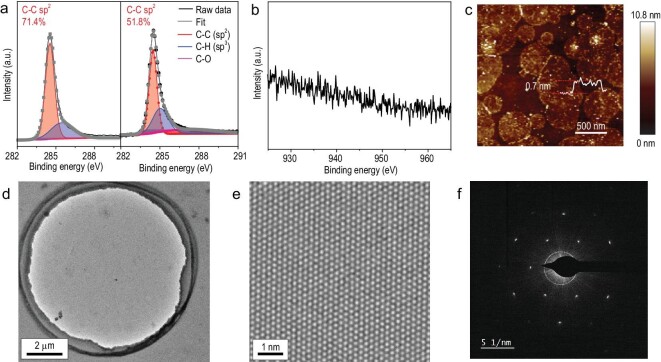
Characterization of the crystal quality and purity of transfer-free graphene grown without/with the presence of Cu(OAc)_2_. (a) XPS C 1s spectra of graphene grown with (left panel) and without (right panel) Cu(OAc)_2_. (b) XPS spectrum in the Cu 2p_3/2_ region indicating that no copper-related chemical species are observed in the sample within the detection limit of XPS. (c) AFM image of a graphene film grown with Cu(OAc)_2_ transferred onto a SiO_2_/Si substrate. (d) TEM image of graphene grown with Cu(OAc)_2_, revealing a clean surface without copper residues. (e) Atomically resolved TEM image of graphene grown with Cu(OAc)_2_ and (f) the corresponding SAED pattern.

X-ray photoelectron spectroscopy (XPS) analysis was performed to probe the surface states during growth. Figure 3a shows the core-level XPS C 1s spectra of graphene grown under different conditions. An asymmetric C 1s peak was fitted with different contributions, with binding energies of 284.5 and 285.4 eV, representing *sp*^2^ and *sp*^3^ carbon signals, respectively [30]. A broad carbon-oxygen bond can also be identified. Notably, Cu(OAc)_2_-facilitated graphene possesses a weaker *sp*^3^ signal, which originates from sufficient decomposition of CH_4_ to inhibit the generation of disordered carbons [31]. Neither the survey spectrum (Fig. S10) nor the high-resolution spectrum for the Cu 2p_3/2_ peak region (Fig. 3b) shows the presence of Cu signals, indicating that there are remaining Cu residues. This further confirmed that introducing a copper catalyst through a metal-containing precursor can effectively control the metal volatilization and address the problem of copper residues. This led to the synthesis of high-quality graphene films with high purity. Meanwhile, atomic force microscopy (AFM) inspection of the transferred graphene film on a SiO_2_/Si substrate also confirmed the monolayer-dominated nature and the absence of metal residues (Fig. 3c). The effective control over metal delivery could be attributed to the optimized amount of Cu clusters released from Cu(OAc)_2_ (Figs S11 and S12). These results are in good agreement with the Raman investigation findings and clearly demonstrate the catalytic merits of the introduced Cu(OAc)_2_. Transmission electron microscopy (TEM) combined with selected area electron diffraction (SAED) was performed to examine the crystal quality of graphene (Fig. 3d–f, Fig. S13). In particular, the atomic-resolved TEM results of the single-layer graphene clearly show the hexagonal atomic configuration of graphene (Fig. 3e). The corresponding diffraction pattern displays a single set of hexagonal electron diffraction patterns (Fig. 3f), indicating the high crystallinity of the grown graphene [32,33].

**Figure 4.**
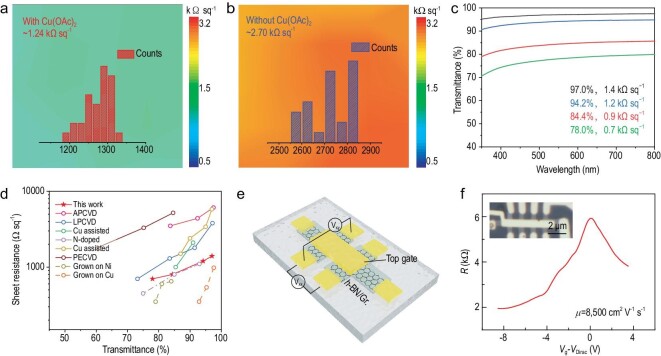
Electrical performances of graphene. (a and b) Sheet resistance maps of graphene (possessing a similar transmittance of 96%) grown with and without Cu(OAc)_2_. Insets: Statistical distribution of the sheet resistance. (c) UV/vis spectra of graphene grown with Cu(OAc)_2_. (d) Statistics of transmittance and sheet resistance of graphene reported in this work and from the literature. (e) Schematic illustration of the fabricated FET device. (f) Resistivity of graphene encapsulated by *h*-BN as a function of difference between the gate voltage (*V*_G_) and Dirac voltage (*V*_Dirac_) at room temperature. Inset: Optical microscopy image of the corresponding device with 1D contact.

A four-point probe method was applied to the as-grown samples to investigate their macroscopic conductivity. Figure 4a and b shows the sheet resistance maps of graphene with and without the presence of Cu(OAc)_2_ during synthesis, respectively. The corresponding histograms of the statistical analysis are displayed in the inset. Maintaining an identical optical transmittance of ∼96% at 550 nm, the graphene sample grown with Cu(OAc)_2_ exhibits a narrow distribution of sheet resistance, affording an average value of 1.24 kΩ sq^−1^, which is markedly lower than that without Cu(OAc)_2_ (2.70 kΩ sq^−1^). Such a sharp reduction in sheet resistance can be ascribed to the reduced defect density and superior crystal quality, which results from the Cu(OAc)_2_ facilitated growth process. Perfect film uniformity was also validated by the homogeneous sheet resistance mapping results.

Optical transparency along with electrical conductivity was accordingly evaluated, which, as a whole, is a key parameter for real applications such as transparent electronics. Figure 4c shows the relationship between the sheet resistance and transmittance of the obtained samples based on Cu(OAc)_2_-facilitated growth, reflecting our capacity to tailor the optical and electrical properties by comprehensively determining the growth conditions. In this respect, the performance of our graphene compares favorably with that reported in the literature, demonstrating that it is one of the best results reported so far for the transfer-free growth of graphene on insulators (Fig. 4d, Table S1) [13,34–40]. It was even found to be comparable to that of nitrogen-doped graphene glass and graphene directly grown on metal substrates. To further highlight the high quality of our Cu(OAc)_2_-facilitated growth of transfer-free graphene films, their carrier mobility was evaluated by fabricating top-gated field-effect transistor devices (Fig. 4e). As for a representative device, a carrier mobility value of 8500 cm^2^ V^−1^ s^−1^ was obtained at room temperature (Fig. 4f), which is much higher than previously reported values for graphene directly grown on insulators (Table S2) [12,41–45].

**Figure 5.**
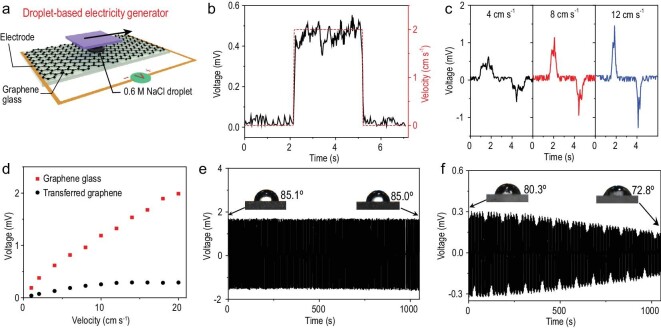
Performance of droplet-based hydrovoltaic electricity generators. (a) Schematic illustration of the device set-up for electricity generation. (b) Typical voltage signal generated by dragging a water droplet on a directly grown graphene/quartz substrate at a constant velocity of 2 cm s^–1^. (c) Voltage signal produced by dragging a water droplet at different velocities. (d) Comparison of voltage signals produced by dragging a droplet on directly grown graphene (red) and transferred graphene (black), respectively. (e and f) Cycling tests of devices based on (e) directly grown graphene and (f) transferred graphene. Insets: Corresponding static contact angles.

Considering the outstanding optical transparency and electrical conductivity of our transfer-free graphene, a hydrovoltaic electricity generator was thus fabricated as a proof-of-concept device for practical applications (Fig. 5) [46]. Figure 5a illustrates the device configuration of the electricity generator based on the graphene/quartz glass substrate. A droplet of 0.6 M NaCl aqueous solution (∼20 μL) was sandwiched between the graphene glass and a 3 × 3 mm^2^ SiO_2_/Si wafer connected to a variable-speed motor (Fig. S14). The droplet could be drawn by the wafer at different velocities, thereby generating a corresponding voltage signal (Fig. S15). When the droplet is dragged from one side to another side at a constant velocity of 2 cm s^–1^, a voltage of 0.5 mV can be responsively produced (Fig. 5b). The voltage generated by the movement of the droplet drops sharply to zero when the droplet ceases to move. When an ionic droplet is laid on the surface of graphene, an electric double layer (EDL) is formed at the solid-liquid interface [46]. Under a static state, there is no potential difference between the two sides of the droplet. As the droplet is dragged forward, the formation of the EDL at the front will draw image charges, while the vanishing of the EDL at the rear will release image charges, thus generating a current flow and electric potential in graphene (Fig. S16).

In comparison with previously reported hydrovoltaic devices based on transferred graphene samples, our directly grown graphene device is free of transfer-related contaminants and possesses a robust contact between graphene and the substrate. As shown in Fig. 5c, the induced voltage varies from 0.5 to 1.5 mV when the velocity shifts from 4 to 12 cm s^–1^. In addition to the velocity of the droplet, the induced voltage is closely related to the direction and the NaCl concentration of the droplet (Figs S17 and S18). Figure 5d shows a comparison of the voltage response against the droplet velocity between our directly grown graphene and the transferred graphene. The transferred graphene was synthesized on Cu foil by CVD and then transferred onto a quartz substrate with the assistance of a polymer. As for the directly grown graphene, the induced voltage displays an almost linear increase with increasing velocity from 2 to 20 cm s^–1^. In contrast, transferred graphene shows a much lower voltage value, where the curve of the voltage response gradually becomes quite flat until the induced voltage no longer increases at 10 cm s^–1^. This could be attributed to the unavoidable damage, corrugation and polymeric contamination caused by the transfer procedure, as well as the inferior adhesive force between the transferred graphene and the substrate. To further highlight the advance of our droplet-based electricity generator, long-term cyclic tests were carried out. As shown in Fig. 5e, the device based on directly grown graphene affords excellent long-term durability with stable voltage output, which can also be reflected by the identical contact angle values before and after cycling (remaining at 85°). Nevertheless, the device based on transferred graphene shows a fading voltage output and a decreased contact angle (from 80.3° to 72.8°), as shown in Fig. 5f. The significant difference between directly grown graphene and transferred graphene could be attributed to the wettability of substrate, which is reflected in the contact angle. The output voltage is closely related to the velocity of droplet movement, as well as the velocity of detachment of droplet at greater contact angle, which means less wettability would lead to larger output voltage. The transferred graphene device shows significant voltage decay because of the decreased water contact angle in the long-time test. The unstable wetting characteristic of the transferred graphene can be ascribed to the unavoidable damage, corrugation and polymeric contaminations in the transfer process. Benefitting from the transfer-free process and stable hydrophobicity characteristic in the air, the directly grown graphene device shows advances in the long-time electrical output. These results collectively reveal the robustness of the transfer-free graphene. Another prototype of a droplet-based hydrovoltaic electricity generator device is shown, in which 0.6 M NaCl solution droplets dropped onto a 70° tilted graphene surface from 15 cm above the contact point under gravity could generate a pulse voltage of 30 mV (Fig. S19). This enhanced voltage could be attributed to the larger velocity of the droplet as well as the larger droplet size when crashing onto the tilted graphene surface [46]. Based on the results, energy harvesting devices such as rain-droplet-driven electricity generators are expected to be realized. Compared to other current hydrovoltaic electricity generator devices based on graphene, the fabricated devices demonstrated higher generated voltages and superior long-term durability (Table S3).

## CONCLUSIONS

In summary, we have demonstrated a copper acetate-facilitated approach for the transfer-free synthesis of graphene films on dielectric substrates, which readily harnesses high quality, high purity and multilayer deficiency. The obtained graphene films exhibited favorable electrical performance with a sheet resistance reaching 1.24 kΩ sq^−1^ at a high optical transmittance of 96% and a carrier mobility reaching 8500 cm^2^ V^−1^ s^−1^, greatly surpassing their directly grown counterparts reported previously. The thus-derived hydrovoltaic electricity generator based on our high-quality directly grown graphene demonstrates impressive voltage output and advanced cyclic stability, which indicates great promise for smart energy harvesting with environmental friendliness and cost effectiveness.

## METHODS

### CVD growth of graphene films on dielectric substrates

Copper acetate-facilitated CVD growth of graphene was performed using two independent heating systems. Prior to the CVD growth of graphene, the substrate (sapphire, quartz) was washed in an ultrasonic bath with deionized water, acetone and isopropanol consecutively, and then dried with a N_2_ blow. Subsequently, the substrate was loaded into a horizontal quartz tube (1- or 3-inch diameter) inside a furnace, with the temperature set to 1030–1080°C, while the Cu(OAc)_2_ powder (50 mg) was loaded with a quartz boat upstream the same horizontal quartz tube with another independent furnace, with the temperature set to 150°C. First, the substrate was heated to 1020–1080°C and annealed in air to eliminate surface contamination. Subsequently, Cu(OAc)_2_ was heated to 150°C to continuously supply metal-containing precursors. Ar was then introduced into the chamber to remove the air, and H_2_ and CH_4_ were introduced into the chamber to initiate the growth of graphene. The graphene films were synthesized using the atmospheric-pressure CVD (APCVD) method. As for the 1-inch system, typical graphene growth was performed with a gas mixture of Ar/H_2_/CH_4_ (200/100/10–25 sccm) for 2–5 hours. After the graphene growth process, the two heating systems were cut off and the sample was cooled to room temperature in an atmosphere of Ar and H_2_. For graphene growth without copper acetate assistance, the process was almost the same, but without the use of Cu(OAc)_2_.

### Theoretical calculation

DFT calculations were performed using the Vienna *ab initio* simulation package (VASP). The exchange-correlation functional was described by the Perdew-Burke-Ernzerhof version of the generalized gradient approximation and the core region by the projector augmented wave method with the cutoff of the plane-wave set as 400 eV. A 1 × 1 × 1 k-point mesh was used for structure relaxation and climbing image nudged elastic band (CI-NEB) calculations. The vacuum layer was larger than 10 Å to avoid interactions with the neighboring images. Using plane-wave-based total energy minimization, all structures were fully relaxed until the force on each atom was less than 0.01 eV/Å. The decomposition energy of CH*_x_* was calculated as the energy difference between CH*_x_* and the case that one H atom is moved far away from CH*_x_*_−1_. The Cu_4_ cluster was the relatively stable structure to participate in the decomposition of CH*_x_*.

### Fabrication of hydrovoltaic devices

For the fabrication of a hydrovoltaic device based on transferred graphene sample, graphene was grown on copper foil by the CVD method and then transferred onto a quartz substrate. Two terminals of the graphene samples were wired with carbon tape to form an ohmic contact, followed by silver epoxy insulation. The fabrication of a hydrovoltaic device based on the directly grown graphene followed identical procedures.

### Voltage measurement

A droplet of 0.6 M NaCl aqueous solution (∼20 μL) was sandwiched between the graphene sample and a 3 × 3 mm^2^ SiO_2_/Si support connected to a variable-speed motor. The droplet can be drawn by the wafer at different velocities. The voltage signal between the two electrodes was recorded in real time using a Keithley DMM6500 multimeter.

## Supplementary Material

nwab169_Supplemental_File
