# Supplementary material for: Copper acetate-facilitated transfer-free growth of high-quality graphene for hydrovoltaic generators
Source: Natl Sci Rev. 2021 Sep 8;9(7):nwab169. doi: 10.1093/nsr/nwab169 (PMC9370374; doi:10.1093/nsr/nwab169)
Supplement: nwab169_Supplemental_File [file nwab169_supplemental_file.pdf]

Supporting Information

Copper Acetate-Facilitated Transfer-Free Growth of High-Quality Graphene for Hydrovoltaic Generators

Jingyuan Shan[1,2†], Sunmiao Fang[3†], Wendong Wang[4†], Wen Zhao[5†], Rui Zhang[4], Bingzhi Liu[6,7], Li Lin[4], Bei Jiang[1], Haina Ci[6,7], Ruojuan Liu[1], Wen Wang[7], Xiaoqin Yang[6], Wenyue Guo[5], Mark H. Rümmeli[6,7], Wanlin Guo[3]*, Jingyu Sun[6,7]* and Zhongfan Liu[1,7]*

[1]Center for Nanochemistry (CNC), Beijing Science and Engineering Center for Nanocarbons, Beijing National Laboratory for Molecular Sciences, College of Chemistry and Molecular Engineering, Peking University, Beijing 100871, P. R. China

[2]Academy for Advanced Interdisciplinary Studies, Peking University, Beijing 100871, P. R. China

[3]Key Laboratory for Intelligent Nano Materials and Devices of the Ministry of Education, State Key Laboratory of Mechanics and Control of Mechanical Structures, Institute of Nanoscience, Nanjing University of Aeronautics and Astronautics, Nanjing 210016, P. R. China

[4]Department of Physics and Astronomy, University of Manchester, Manchester M13 9PL, UK

[5]School of Materials Science and Engineering, China University of Petroleum (East China), Qingdao 266580, P. R. China

[6]College of Energy, Soochow Institute for Energy and Materials InnovationS (SIEMIS), Jiangsu Provincial Key Laboratory for Advanced Carbon Materials and Wearable Energy Technologies, Soochow University, Suzhou 215006, P. R. China

[7]Beijing Graphene Institute (BGI), Beijing 100095, P. R. China

*Corresponding Author: zfliu@pku.edu.cn (Z. F. Liu); sunjy86@suda.edu.cn (J. Y. Sun); wlguo@nuaa.edu.cn (W. L. Guo)

†These authors contributed equally to this work.

Methods

Characterizations. Optical microscopy (OM) images were obtained with Olympus BX51 microscopy. Raman spectra were acquired from LabRAM HR-800 (Horiba, LabRAM HR Evolution, 532 nm laser wavelength, ×100 objective). Scanning electron microscopy (SEM) images were taken from Thermo Fisher Scientific, FEI Quattro S (acceleration voltage 1-30 kV). The atomically resolved TEM investigations and selected area electron diffraction (SAED) patterns of graphene were conducted on a FEI Titan Themis TEM equipped with a monochromator and a C_s corrector for the primary objective lens. The electron acceleration voltage was 80 kV. The UV-vis transmittance spectra were obtained with Perkin-Elmer Lambda 950 spectrophotometer. The sheet resistances characterizations were performed by CDE, ResMap 178 four-probe resistance measuring meter. Atomic force microscope (AFM) characterization was carried out on a Bruker dimension icon with ScanAsyst mode. The element analysis was performed by X-ray photoelectron spectroscopy (XPS) (Kratos Analytical AXIS-Ultra with monochromatic Al Kα X-ray).

Fabrication of mobility measurement devices. The mobility measurement devices were made from the monolayer graphene grown on quartz via CVD method. The graphene was protected by relatively thick (~ 80 nm) crystal of hexagonal boron nitride (*h*-BN), which was transferred by dry-peel technique using PDMS/PPC stack. After this, the devices were fabricated using electron-beam lithography and standard etching procedures for top gate deposition, 1D contacts electrodes (3 nm Cr and 80 nm Au), and Hall bar shaping. The measured Hall bar was 500 nm width and 500 nm length between each bar aimed to avoid the inhomogeneity caused by the graphene domain wall and contamination bubbles.

Mobility measurement. The electrical properties of the fabricated devices were characterized with the conventional lock-in technique. An AC current I_{ds} with a root mean square amplitude of 1 μA at 23.33 Hz was applied between the source and drain terminals. Meanwhile, the four-point longitude voltage drop V_{xx} and transverse voltage drop V_{xy} were measured with lock-in amplifiers. The charge density tuning in the graphene channel is achieved by applying different back gate voltage V_g to the Si substrate. To eliminate the negative effect of oxygen and water in air on the device performance, the device was tested either in Argon inertial environment (glovebox at the temperature of 300 K) or vacuum environment (cryostat at the temperature of <100 K).

The longitude resistivity ρ_{xx} can be calculated from $\rho_{xx} = R_{xx}*W/L$, where W is the width of the conducting channel, L is the length of the channel between the probed contacts, and R_{xx} is the longitude resistance $R_{xx}=V_{xx}/I_{ds}$, and hence the longitude conductivity σ_{xx} can be obtained via $\sigma_{xx} =1/\rho_{xx}$. The Hall carrier density n was determined by $n=B/(\mathrm{e}R_{xy})$, where e is the elementary charge and R_{xy} is the Hall resistance $R_{xy}=V_{xy}/I_{ds}$ at the corresponding perpendicular magnetic field B. Based on the Drude model, mobility $\mu=\sigma_{xx}/(n\mathrm{e})$ can be estimated from the slop of linear regions of σ_{xx} versus n plots.

Supporting Figures and Tables

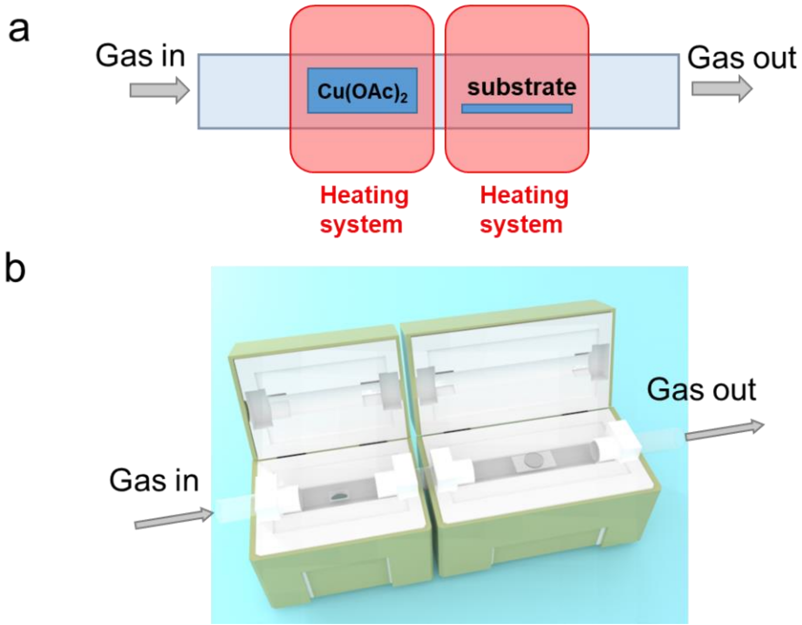

Figure S1. Schematics of the homemade CVD system with two independent heating systems for the copper acetate-facilitated CVD growth of graphene films.

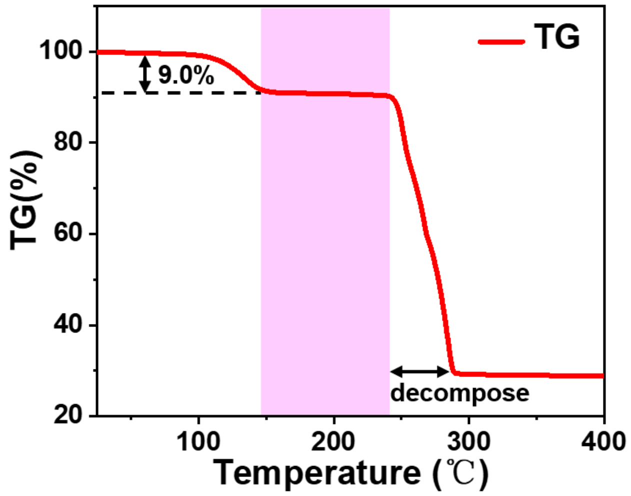

Figure S2. TG-MS spectra of the $Cu(OAc)_2$. The $Cu(OAc)_2$ volatilized between the temperature from 140 ℃ to 240 ℃ and then decomposed from 240 ℃ .

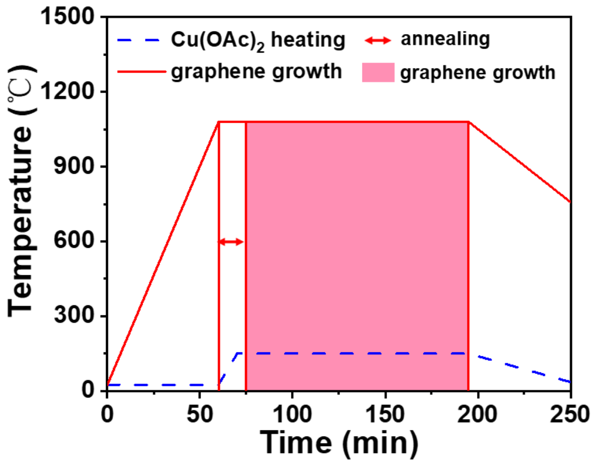

Figure S3. CVD process for the preparation of graphene film with homemade CVD system (red for graphene growth and blue for $Cu(OAc)_2$ heating).

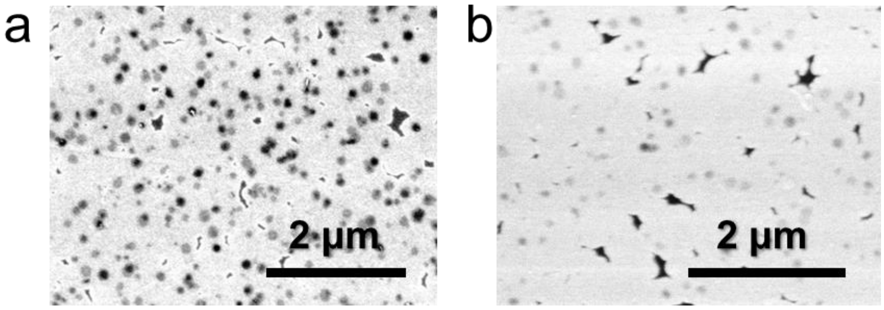

Figure S4. Representative SEM images of graphene film (a) without $Cu(OAc)_2$ and (b) with $Cu(OAc)_2$.

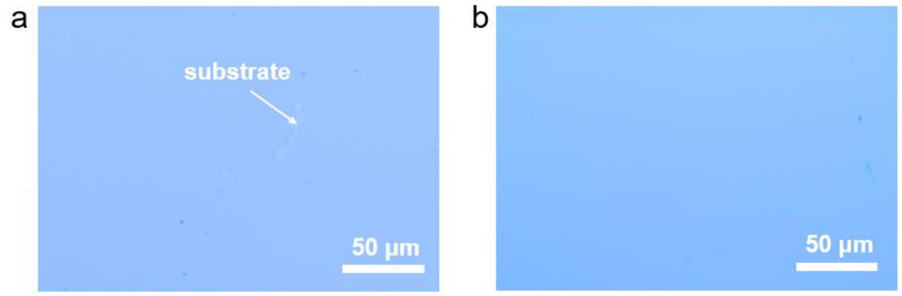

Figure S5. Optical images of as-grown graphene transferred onto SiO_2/Si substrate (a) with $Cu(OAc)_2$ and (b) without $Cu(OAc)_2$.

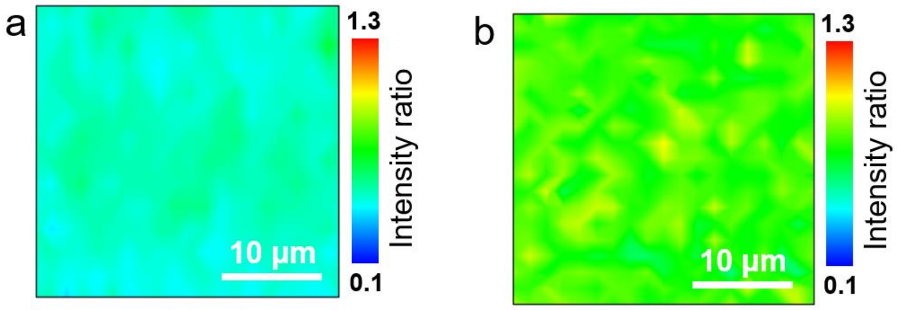

Figure S6. Raman mappings of I_D/I_G of graphene film (a) with $Cu(OAc)_2$ and (b) without $Cu(OAc)_2$.

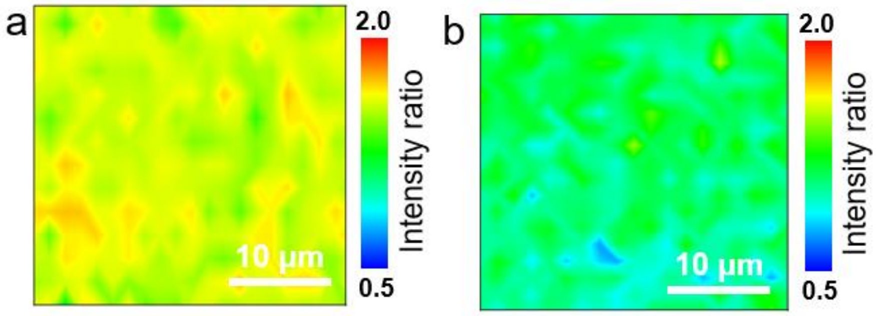

Figure S7. Raman mappings of I_{2D}/I_G of graphene film (a) with $Cu(OAc)_2$ and (b) without $Cu(OAc)_2$.

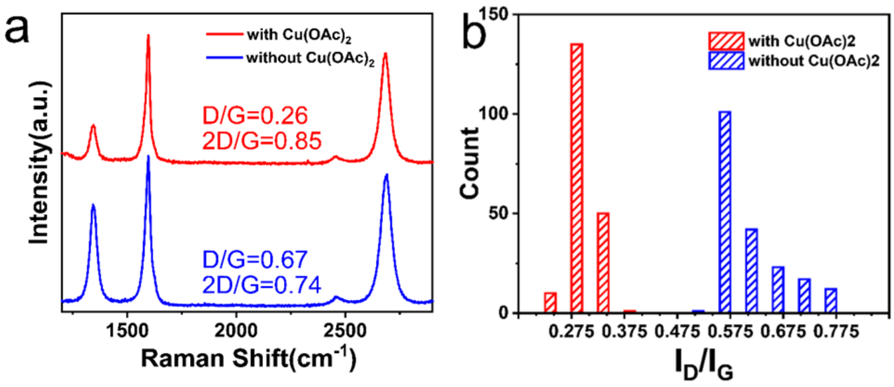

Figure S8. (a) Representative Raman spectra of graphene film directly grown on quartz before transfer process with $Cu(OAc)_2$ (red) and without $Cu(OAc)_2$ (blue); (b) The histograms acquired over a survey area of 30 × 30 μm^2 represent significant reduction of I_D/I_G of graphene film with $Cu(OAc)_2$ (red) and without $Cu(OAc)_2$ (blue).

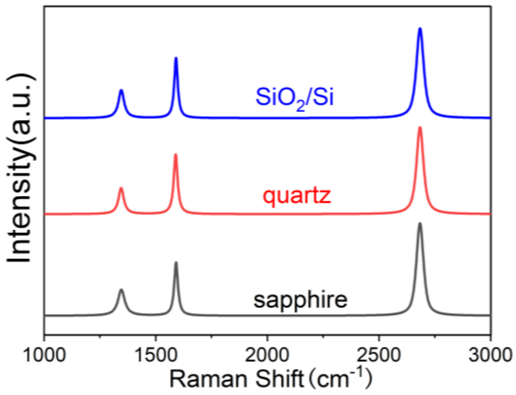

Figure S9. Representative Raman spectra of graphene film directly grown on sapphire, quartz and SiO_2/Si substrates with $Cu(OAc)_2$.

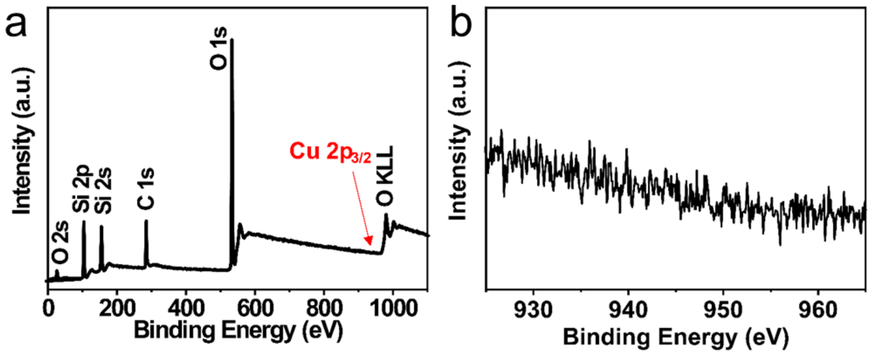

Figure S10. (a) X-ray photoelectron spectroscopy (XPS) survey spectrum. (b) XPS spectrum in Cu $2p_{3/2}$ region indicating that any copper-related chemical species are not observed within the detection limit of XPS from the sample.

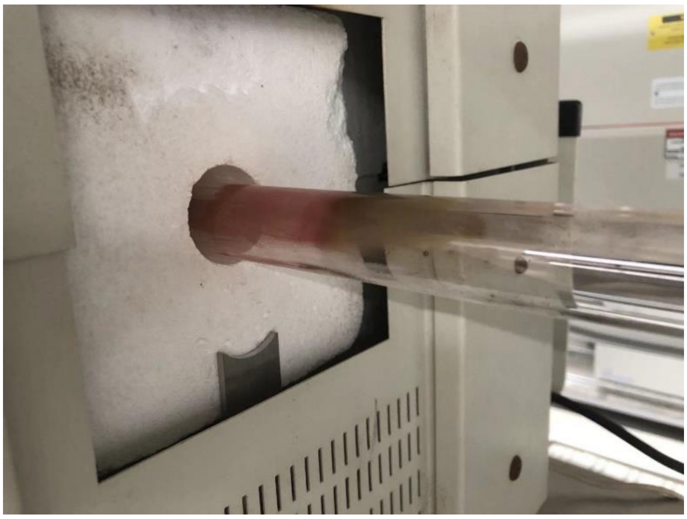

Figure S11. The as-obtained copper clusters deposited out of the furnace at the downstream of the system. The copper clusters generated from $Cu(OAc)_2$ in high temperature zone and participated in the catalytic process of graphene growth and then deposited in low temperature zone. Hence there won’t be metal residues from the flat substrate in high temperature zone

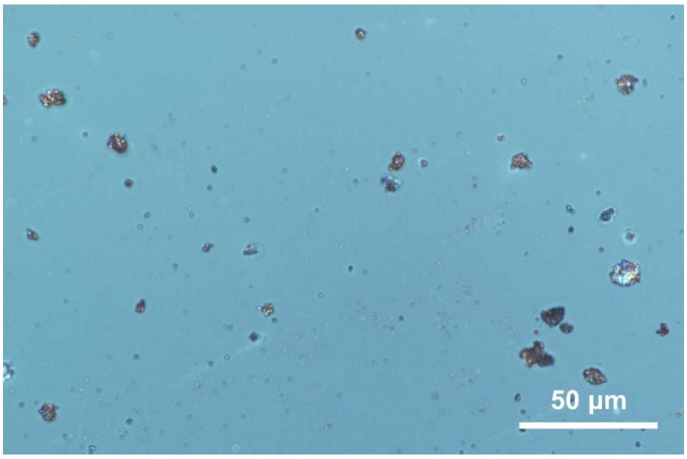

Figure S12. Optical image of as-grown graphene transferred onto SiO_2/Si substrate with $Cu(OAc)_2$ heated at the temperature of 180 ℃. By heating the $Cu(OAc)_2$ beyond the temperature of 150 ℃, there would generate excessive copper clusters and then deposited on the substrate.

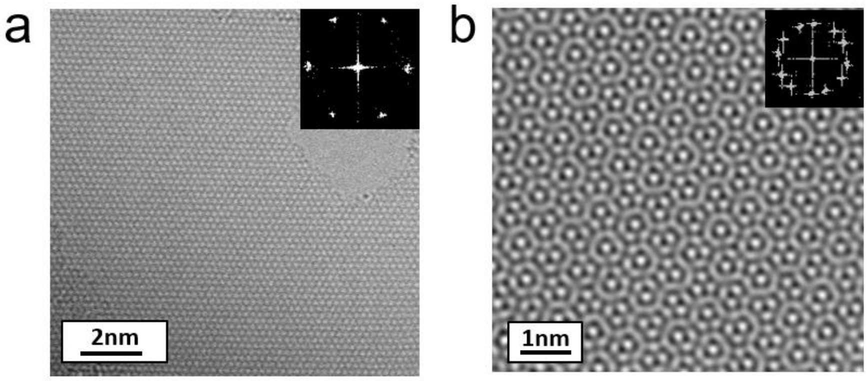

Figure S13. (a) TEM image of graphene grown with $Cu(OAc)_2$, revealing the monolayer dominated graphene film without copper residues. (b) Atomically resolved TEM image showing the coexistence of bilayer regions. Inset: The corresponding FFT pattern. The growth of partial bi-layer graphene is unavoidable.

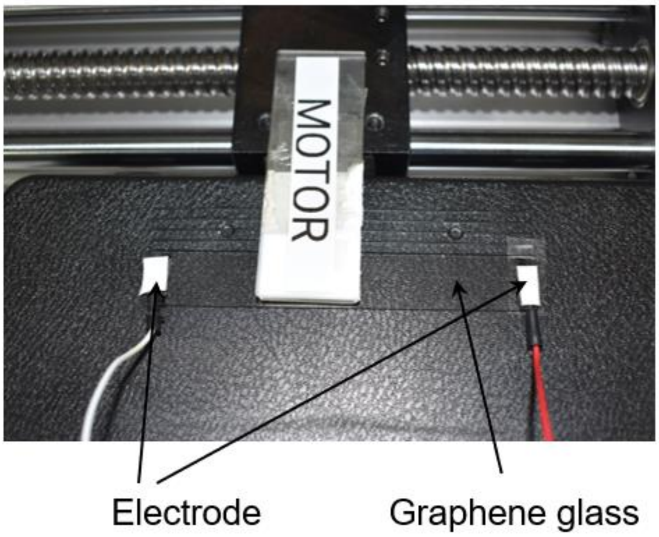

Figure S14. Photograph of the droplet-based electricity generator device, with a motor drawing the droplet by a SiO_2/Si wafer to generate voltage signal.

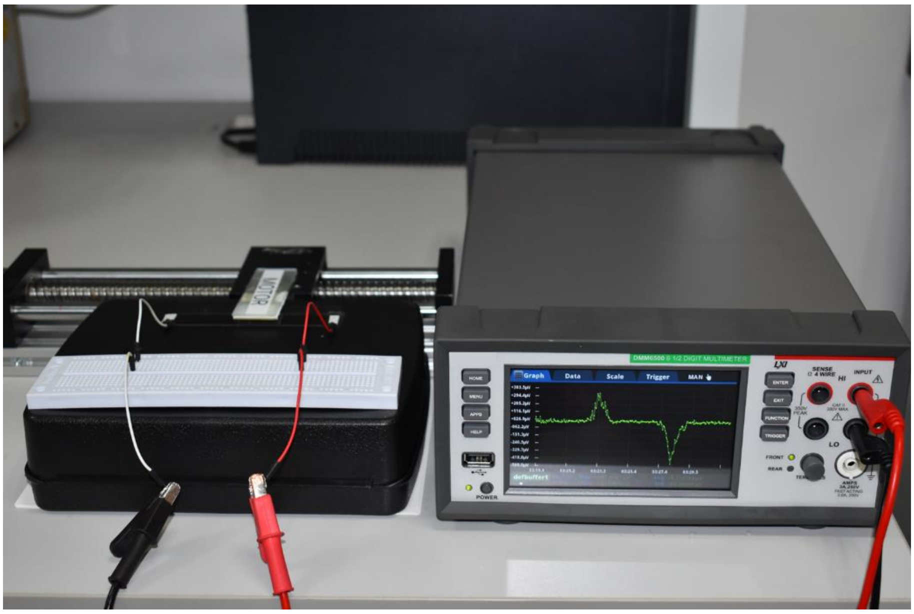

Figure S15. Photograph of the measurement of the droplet-based electricity generator device. When the motor draws the droplet by a SiO_2/Si wafer, there will be an electric signal shown on the multimeter.

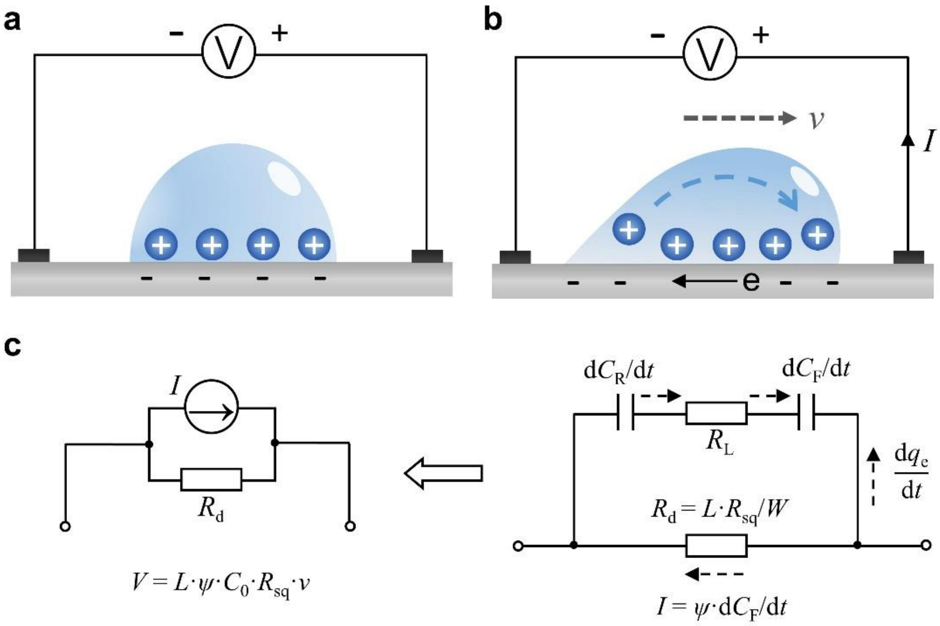

Figure S16. Mechanism for the droplet-induced voltage on the graphene. (a, b) Schematic illustration of the charge distribution in a static (a) and moving droplet (b) on the graphene. The direction of generated voltage and current is also presented. (c) Equivalent (right) and simplified circuit (left) for the system. C_F and C_R represent the two pseudocapacitors forming at the front and rear end of the graphene-droplet interface, respectively. R_L and R_d denote the resistance of the droplet and the graphene. L and W are the length and width of the droplet.

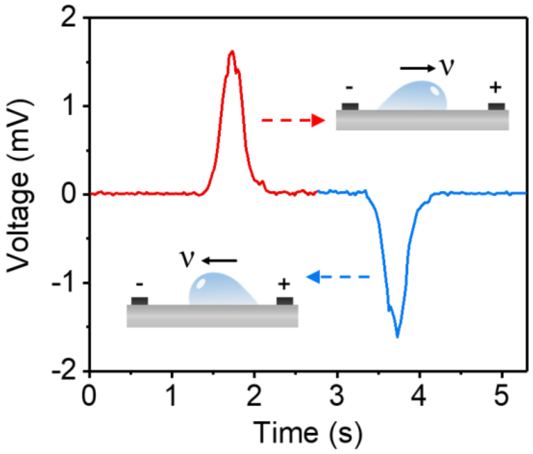

Figure S17. Voltage signal produced by dragging a 0.6 M NaCl droplet on based on as prepared graphene/quartz glass substrate in different directions at the velocity of 20 cm s^{-1}. Insets: Corresponding schematic illustration of moving droplet in different directions.

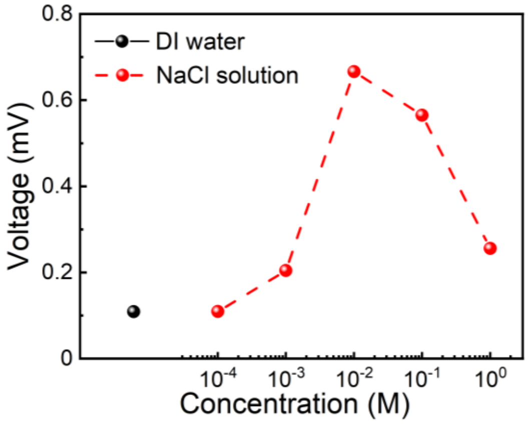

Figure S18. Voltage signals produced by dragging a droplet on as prepared graphene/quartz glass substrate with different NaCl concentrations at the velocity of 6 cm s^{-1}.

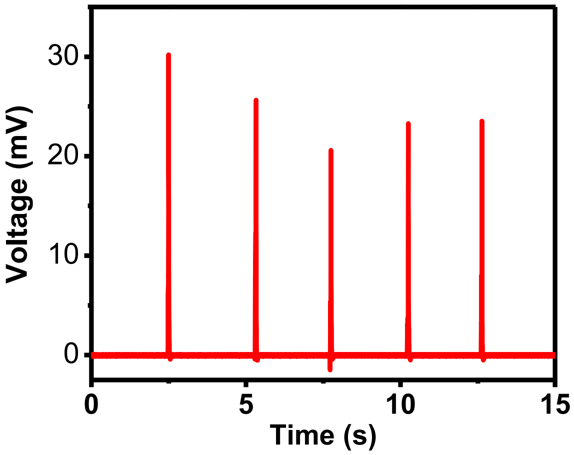

Figure S19. Voltage produced by dropping droplets of 0.6 M NaCl solution onto graphene glass substrate at an angle of 70 °. Dropping droplets of NaCl solution onto a tilted graphene glass could generate a pulse voltage of 20-30 mV. The enhanced voltage could be attributed to the improved velocity of the droplet as well as the greater droplet size when crashed onto the tilted graphene surface [1]. Based on the result, energy harvesting devices such as rainwater droplet driven electricity generators are expected to be realized.

Table S1. Comparison of visible-light transmittance (550 nm) and sheet resistance from this work and others in the literature for graphene.

	Method	Substrate	Precursor	Temperature (℃)	Transmittance	Sheet Resistance ($\Omega\ sq^{-1}$)	Ref.
**1**	$Cu(OAc)_2$ facilitated	quartz	CH_4	1080	96%	1240	This work
**2**	APCVD	quartz	CH_4	1020	97.5%	6100	[2]
**3**	LPCVD	quartz	ethanol	1100	97.2%	3800	[3]
**4**	Cu assisted	quartz	CH_4	1050	91%	2100	[4]
**5**	Cu assisted	SiO_2/Si	CH_4	1000	97%	5899	[5]
**6**	N-doped	quartz	CH_4, methylamine	1040	93%	1100	[6]
**7**	PECVD	quartz	CH_4	600	84.6%	5200	[7]
**8**	Grown on Ni	Ni	CH_4	1000	84%	650	[8]
**9**	Grown on Cu	Cu	CH_4	1000	97.6%	980	[9]

Table S2. Comparison of FET hole carrier mobility from this work and others in the literature for graphene over dielectric substrates.

No.	Substrate	FET hole carrier mobility ($cm^2 V^{-1} s^{-1}$)	Type	Ref.
1	quartz	8500	Top-gate	This work
2	SiO_2/Si	2300	Back-gate	[10]
3	SiO_2/Si	800	Back-gate	[11]
4	SiO_2/Si	550	Top-gate	[12]
5	SiO_2/Si	760	Back-gate	[13]
6	SiO_2/Si	4000	Back-gate	[14]
7	SiO_2/Si	1510	Back-gate	[15]
8	quartz	710	Back-gate	[2]

Table S3. Comparison of the hydrovoltaic generator from this work and others in the literature based on graphene.

No.	Type	Material	Method	Substrate	Voltage	Cycle index	Ref.
1	Moving droplet	Graphene film	CVD (direct growth)	Quartz	0.5 mV	333	This work
2	Dropping droplet	Graphene film	CVD (direct growth)	Quartz	30 mV	/	This work
3	Moving droplet	Graphene film	CVD (transfer)	PET	0.15 mV	/	[1]
4	Dropping droplet	Graphene film	CVD (transfer)	PET	25 mV	/	[1]
5	Dropping droplet	GO film	Electrophoresis	ITO/Glass /FTO	62 μV	45	[16]
6	Dropping droplet	G-CB/PTFE	Wet chemistry	FTO glass	77.52 μV	/	[17]
7	Moving droplet	Graphene film	CVD (transfer)	PET	0.2 mV	/	[18]
8	Waving	Graphene film	CVD (transfer)	PET	3.3 mV	/	[19]
9	Flowing	Graphene film	CVD (transfer)	SiO_2/Si	30 mV	/	[20]

Supporting References

1. Yin J, Li X and Yu J *et al*. Generating Electricity by Moving a Droplet of Ionic Liquid Along Graphene. *Nat Nanotechnol* 2014; **9**: 378-83.

2. Sun J, Chen Y and Priydarshi M K *et al*. Direct Chemical Vapor Deposition-Derived Graphene Glasses Targeting Wide Ranged Applications. *Nano Lett.* 2015; **15**: 5846-54.

3. Chen X, Chen Z and Jiang W *et al*. Fast Growth and Broad Applications of 25-Inch Uniform Graphene Glass. *Adv. Mater.* 2017; **29**: 1603428.

4. Sun J, Chen Z and Yuan L *et al*. Direct Chemical-Vapor-Deposition-Fabricated, Large-Scale Graphene Glass with High Carrier Mobility and Uniformity for Touch Panel Applications. *ACS Nano* 2016; **10**: 11136-44.

5. Teng PY, Lu CC and Akiyama-Hasegawa K *et al*. Remote Catalyzation for Direct Formation of Graphene Layers on Oxides. *Nano Lett.* 2012; **12**: 1379-84.

6. Cui L, Chen X and Liu B *et al*. Highly Conductive Nitrogen-Doped Graphene Grown on Glass toward Electrochromic Applications. *ACS Appl. Mater. Inter.* 2018; **10**: 32622-30.

7. Sun J, Chen Y and Cai X *et al*. Direct Low-Temperature Synthesis of Graphene on Various Glasses by Plasma-Enhanced Chemical Vapor Deposition for Versatile, Cost-Effective Electrodes. *Nano Res.* 2015; **8**: 3496-504.

8. Kim KS, Zhao Y and Jang H *et al*. Large-Scale Pattern Growth of Graphene Films for Stretchable Transparent Electrodes. *Nature* 2009; **457**: 706-10.

9. Suk J W, Kitt A and Magnuson CW *et al*. Transfer of CVD-Grown Monolayer Graphene onto Arbitrary Substrates. *ACS Nano* 2011; **5**: 6916-24.

10. Mishra N, Forti S and Fabbri F *et al*. Wafer-Scale Synthesis of Graphene on Sapphire: Toward Fab-Compatible Graphene. *Small* 2019; **15**: 1904906.

11. Kim H, Song I and Park C *et al*. Copper-Vapor-Assisted Chemical Vapor Deposition for High-Quality and Metal-Free Single-Layer Graphene on Amorphous SiO_2 Substrate. *ACS Nano* 2013; **7**: 6575-82.

12. Song I, Park Y and Cho H *et al*. Transfer-Free, Large-Scale Growth of High-Quality Graphene on Insulating Substrate by Physical Contact of Copper Foil. *Angew. Chem. Int. Ed.* 2018; 57: 15374-8.

13. Pang J, Mendes RG and Wrobel PS *et al*. Self-Terminating Confinement Approach for

Large-Area Uniform Monolayer Graphene Directly over Si/SiO_X by Chemical Vapor Deposition. *ACS Nano* 2017; **11**: 1946-56.

14. Wang H, Xue X and Jiang Q *et al*. Primary Nucleation-Dominated Chemical Vapor Deposition Growth for Uniform Graphene Monolayers on Dielectric Substrate. *J. Am. Chem. Soc.* 2019; **141**: 11004-8.

15. Chen J, Guo Y and Wen Y *et al*. Two-Stage Metal-Catalyst-Free Growth of High-Quality Polycrystalline Graphene Films on Silicon Nitride Substrates. *Adv. Mater.* 2013; **25**: 992-7.

16. Zhang Y, Tang Q and He B *et al*. Graphene Enabled All-Weather Solar Cells for Electricity Harvest from Sun and Rain. *J. Mater. Chem. A* 2016; **4**: 13235-41.

17. Tang Q, Zhang H and He B *et al*. An All-Weather Solar Cell That Can Harvest Energy from Sunlight and Rain. *Nano Energy* 2016; **30**: 818-24.

18. Zhong H, Li X and Wu Z *et al*. Two Dimensional Graphene Nanogenerator by Coulomb Dragging: Moving Van Der Waals Heterostructure. *Appl. Phys. Lett.* 2015; **106**: 243903.

19. Yin J, Zhang Z and Li X *et al*. Waving Potential in Graphene. *Nat. Commun.* 2014; **5**: 3582.

20. Dhiman P, Yavari F and Mi X *et al*. Harvesting Energy from Water Flow over Graphene. *Nano Lett.* 2011; **11**: 3123-7.
